# Intraspecies multiple chromosomal variations including rare tandem fusion in the Russian Far Eastern endemic evoron vole *Alexandromysevoronensis* (Rodentia, Arvicolinae)

**DOI:** 10.3897/compcytogen.v15.i4.67112

**Published:** 2021-11-22

**Authors:** Irina V. Kartavtseva, Irina N. Sheremetyeva, Marina V. Pavlenko

**Affiliations:** 1 Federal Scientific Center of the East Asia Terrestrial Biodiversity Far Eastern Branch of Russian Academy of Sciences, Vladivostok 690022, Russia

**Keywords:** Chromosomal races, chromosomal rearrangements, polymorphism, Robertsonian translocation, tandem fusion

## Abstract

The vole *Alexandromysevoronensis* (Kovalskaya et Sokolov, 1980) with its two chromosomal races, “Evoron” (2n = 38–41, NF = 54–59) and “Argi” (2n = 34, 36, 37, NF = 51–56) is the endemic vole found in the Russian Far East. For the “Argi” chromosomal race, individuals from two isolated populations in mountain regions were investigated here for the first time using GTG-, GTC-, NOR methods. In the area under study, 8 new karyotype variants have been registered. The karyotype with 2n = 34 has a rare tandem fusion of three autosomes: two biarmed (Mev6 and Mev7) and one acrocentric (Mev14) to form a large biarmed chromosome (Mev6/7/14), all of which reveal a heterozygous state.

For *A.evoronensis*, the variation in the number of chromosomes exceeded the known estimate of 2n = 34, 36 and amounted to 2n = 34, 36, 38–41. The combination of all the variations of chromosomes for the species made it possible to describe 20 variants of the *A.evoronensis* karyotype, with 11 chromosomes being involved in multiple structural rearrangements. In the “Evoron” chromosomal race 4 chromosomes (Mev1, Mev4, Mev17, and Mev18) and in the “Argi” chromosomal race 9 chromosomes (Mev6, Mev7, Mev14, Mev13, Mev11, Mev15, Mev17, Mev18, and Mev19) were observed. Tandem and Robertsonian rearrangements (Mev17/18 and Mev17.18) were revealed in both chromosomal races “Evoron” and “Argi”.

## Introduction

With evolutionary processes underway, structural chromosomal rearrangements (fusion) could be of great importance (White 1973; King 1993; Ferguson-Smith and Trifonov 2007; Bakloushinskaya 2016; Dobigny et al. 2017). Studies of the tandem rearrangements in animal speciation are of great interest (Huang et al. 2006; Kulemzina et al. 2009; Swier et al. 2009; Bulatova et al. 2020), since it has been shown that translocations and tandem fusions cause transformation of karyotypes in many groups of mammals (White 1973; Elder 1980; Huang et al.1980; Elder and Hsu 1988; King 1993).

Voles of the genus *Microtus* (Schrank, 1798) sensu lato represent one of the groups in which speciation processes are accompanied by intense chromosomal rearrangements (Modi 1987; Meyer et al. 1996; Mazurok et al. 2001; Sitnikova et al. 2007; Lemskaya et al. 2010, 2015; Romanenko et al. 2018). Some species of the genus *Microtus* have karyotypic polymorphism with structural rearrangements (Zagorodnyuk 1990). Numerous observations described nine chromosomal races of vole endemic to the Balkan region in M. (Terricola) thomasi (Barrett-Hamilton, 1903), and two in M. (T.) atticus (Miller, 1910) (Giagia-Athanasopoulou et al. 1995; Giagia-Athanasopoulou and Stamatopoulos 1997; Rovatsos et al. 2021). The variation in the number of chromosomes (2n = 38, 40–44) among the chromosomal races of M. (Terricola) thomasi can probably be attributed to the Robertsonian translocations and tandem fusions (Rovatsos et al. 2011, 2017). Eleven karyomorphs with different chromosomal numbers (2n = 54–52, 46–43, 42 “A”, 42 “B”, 40, 38) and with a stable number of chromosomal arms (NF = 58) have been described for the endemic cryptic vole species in the Caucasian Region M. (T.) daghestanicus (Schidlovskii, 1919) (the Robertsonian fan) (Lyapunova et al. 1988; Akhverdyan et al. 1992). For two species of the related genus *Alexandromys* Ognev, 1914, inhabiting northeastern Asia – *Alexandromysmaximowiczii* (Schrank, 1859) and *A.evoronensis* (Kovalskaya et Sokolov, 1980) a polymorphism has emerged due to structural rearrangements of chromosomes, including Robertsonian translocations and tandem fusions (Meyer et al. 1996; Kartavtseva et al. 2008, 2021). The *A.maximowiczii* has four chromosomal polymorphic forms (2n = 36–42) in Transbaikalia, the Russian Far East, and Mongolia (Kartavtseva et al. 2008, 2013). *A.evoronensis* has two chromosomal races in isolated populations in the mountainous regions of the Russian Far East (Kartavtseva et al. 2018).

**Figure 1.**
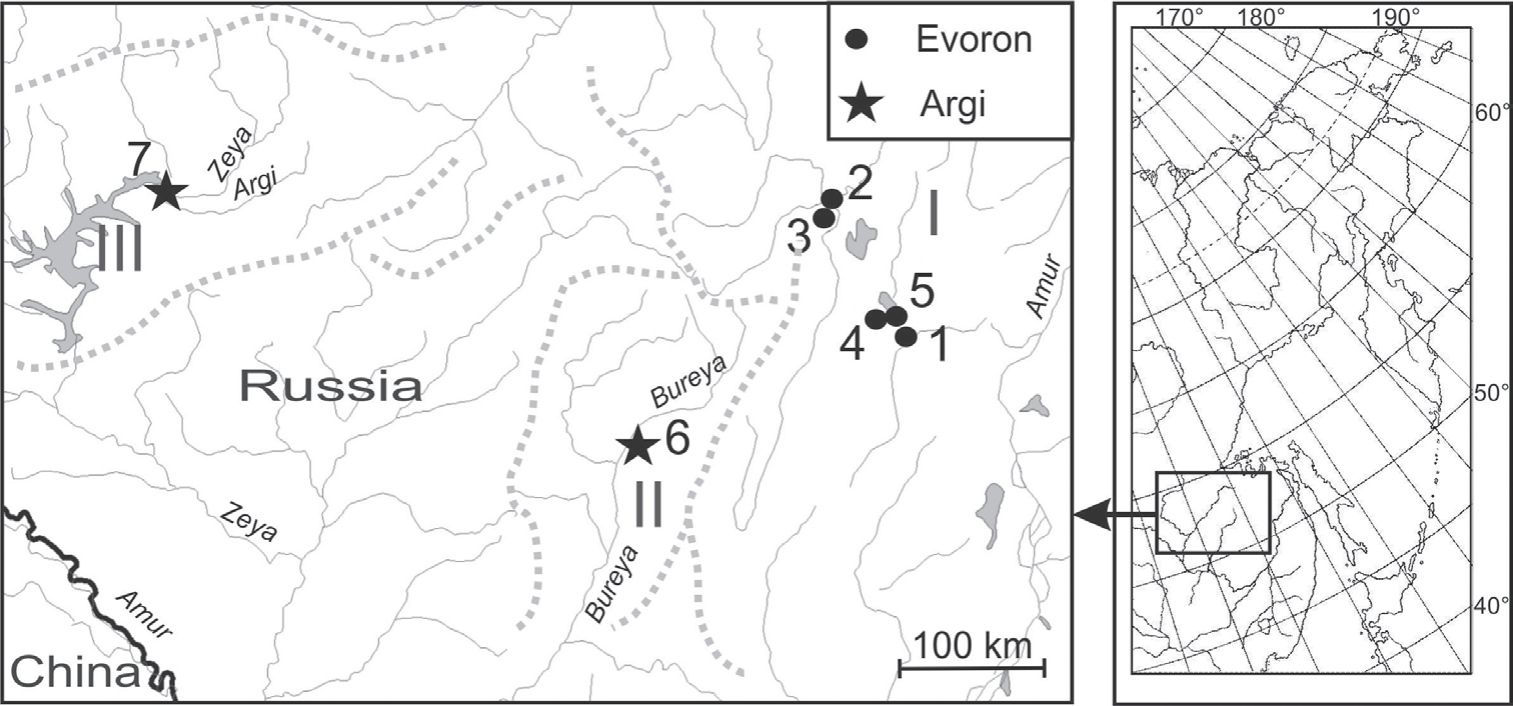
The map showing the collection localities of the *Alexandromysevoronensis* specimens was used in this study with the following legend: black circles stand for “Evoron” chromosomal race Nos. 1–5 according to Kartavtseva et al. 2021; black stars – for “Argi” chromosomal race Nos. 6–7 that are discussed in the material. Intermountain regions of the South of the Russian Far East: I – Evoron-Chukchagirskaya Lowland; II – Verkhnebureinskaya Depression; III – Verkhnezeiskaya Plain. Rows of dotted lines indicate mountain ranges.

The Evoron vole *A.evoronensis* is the endemic vole species found in the intermountain landscape of the southern Russian Far East (Fig. 1). It inhabits the Evoron-Chukchagir lowland, the Upper Zeya Plain (Sheremetyeva et al. 2017a), and the Upper Bureya Depression (Sheremetyeva et al. 2017b). Their description was based on the mtDNA data. The Evoron voles of the Evoron-Chukchagir lowland, with the maximum number of chromosomes for the species (2n = 38–41) (Kartavtseva et al. 2021), belong to the “Evoron” chromosomal race, as they were the first to be found on the shores of Lake Evoron (Kovalskaya and Sokolov 1980). Voles with the minimum number of chromosomes for the species (36 and 37) were assigned to the “Argi” chromosomal race to be later named after the Argi River, the Zeya River tributary (Kartavtseva et al. 2018).

We have confirmed the chromosomal polymorphism of *A.evoronensis* (Kartavtseva et al. 2021) which had been previously discovered (2n = 38–40) and described by Rajabli and Sablina (Meyer et al. 1996). Analysis of differentially stained chromosomes (GTG-, C-, NOR) of voles of the “Evoron” chromosomal race (Kartavtseva et al. 2021) allowed us for the first time to describe 2n = 41 and twelve karyotype variants: two with 2n = 41, six with 2n = 40, three with 2n = 39, and one with 2n = 38. The most impressive was the tandem telomere-telomere fusion (TTel) of two metacentric No 1 and No 4 of autosomes (named Mev1 and Mev4, accordingly; see Kartavtseva et al. 2021), as a result of inactivation of the centromere in one of the metacentric forms a large biarmed element (Mev1/4).

Three variants of acrocentric chromosomes fusions and one variant of metacentric chromosomes fusion were first suggested using the G-banding of the chromosomes of voles from Lake Evoron shores without numbers for all pairs (Meyer et al. 1996). We have confirmed the data by studying the voles of Chukchagir and Evoron lakes using GTG-, C- and NOR methods. We also assigned numbers to these chromosomes (Mev17 and Mev18) and indicated that Mev17 and Mev20 carry NORs (Kartavtseva et al. 2021). The tandem fusion of telomere-centromere (TCen) of two acrocentrics (Mev17 and Mev18) were shown to form chromosomes with distinct morphology, acrocentric (Mev17/18 A) by TCen and metacentric (Mev17/18 M) by TCen. The metacentric variant of the Mev17.18 (Rb translocation) also emerged after the centric fusion of the Mev17 and Mev18.

The variability in the number of chromosome arms may be related to the centromere positions in two pairs of autosomes (Mev8 and Mev13). All the detected chromosomal rearrangements of the Evoron vole karyotype of the “Evoron” chromosomal race were found in both homozygous and heterozygous states. The tandem fusion (Mev1/4) of two metacentric autosomes Mev1 and Mev4 was taken as a marker for the vole karyotype of the Evoron – Chukchagir lowland population (Kartavtseva et al. 2021). Voles of laboratory breeding carrying such rearrangement did not reveal any effect on the fertility and viability of the offspring. The kind of rearrangements which created new karyotype variants of the “Argi” chromosomal race in two isolated populations from the Upper Zeya Plain and the Upper Bureya Depression remains unclear.

This work focuses on studying structural rearrangements in two isolated populations of the “Argi” chromosomal race. A comparative analysis of chromosomal rearrangements in the “Argi” and “Evoron” chromosomal races using GTG-, GTC-, NOR methods was done, which revealed the similarity and difference of the two chromosomal races.

## Material and methods

A total of 17 individuals of *A.evoronensis* (chromosomal race “Argi”) from two populations of the Russian Far East and 26 laboratory-bred voles were studied. Since the voles from five local populations (Nos. 1–5) of the “Evoron” chromosomal race have been studied before (Sheremetyeva et al. 2017a, b), this work continues numbering populations as Nos. 6–7, see Fig. 1. When using animals in research, all applicable international, national and institutional ethical standards have been met.

The voles studied were assigned a double number: zoological number/tissue sample number of mt DNA. The numbers were provided to link the present study with the previously published mt DNA data (Sheremetyeva et al. 2017a, b). From population No. 6 in the Urgal River valley in the Verkhnebureinskaya depression of the Khabarovsk Territory, eight individuals were captured in August 2019, about 40 km south-west of the Chegdomyn village (51°05'54.49"N, 132°33'04.79"E) and near the village, located on the eastern bank of the Bureya River (51°07'34.15"N, 132°31'11.55"E). Females received the following identifying numbers: 4549 / 101–19, 4550 / 102–19, 4553 / 105–19, 4556 / 107–19, 4557 / 108–19 and 4567 / 121–19; respectively, male numbers were 4548 / 100–19 and 4554 / 106–19. The karyotypes of four laboratory animals from one pair of voles (without karyotyping) were also examined.

From population No. 7 in the Argi River valley of the Upper Zeya Plain in the Amur Region (54°40'10.62"N, 129°06'39.73"E), 9 individuals were caught in July 2015; females received the following identifying numbers: 3992 / 22–15 and 3997 / 27–15; male numbers were 3950 / 4–15, 3991 / 21–15, 3993 / 23–15, 3994 / 24–15, 3995 / 25–15, 3996 / 26–15, 3999 / 29–15. The karyotypes of 22 laboratory-bred individuals from parental individuals – female 3992 and males 3995, 3996 with 2n = 36 were also studied.

Chromosome suspensions were prepared from femoral bone marrow cells using the standard method (Ford and Hamerton 1956). The homology of chromosome pairs was detected using the GTG method (Seabright 1971). The Sumner method (1972) with a slight modification, i.e., without the preparation’s pretreatment in hydrochloric acid, was used to reveal C-heterochromatic blocks. Nucleolar organizer staining (NOR) was performed according to the Miinke and Schmiady (1979) method, with a modification that eliminates the pretreatment with formic acid. We inferred the fundamental number (chromosome arm number, NF) for females based on the absence of the acrocentric Y and the presence of two metacentric X chromosomes (Table 1).

**Table 1.** Chromosomal characteristics of karyotype variants and the scheme of variable pairs of autosomes of *Alexandromysevoronensis* from two localities – the Verkhnebureinskaya Depression (No. 6) and the Verkhnezeiskaya Plain (No. 7).

		Autosome number		Number and morphology of autosome pairs					Population	
									No. 6	No. 7
2n	NF	M	A+ St	13/15 (v) or 13.15 (x)	17/18 (v) or 17.18 (x)	7	10	6/7/14	Zoological number of animals	
37a	55	16	19	x v	x x	x x	v v		4554 m	
37b	55	16	19	x v	x v	x x	v v		4556 f	
36a	54	16	18	**v v**	x x	x x	v v		Lb	3992 f, 3950 m, 3991 m, Lb
36b	56	18	16	**x x**	x x	x x	v v		Lb, 4548 m, 4550 f, 4557 f,	Lb
36c	55	17	17	**xv**	xx	xx	vv		4549 f	3994 m, 3997 f, 3999 m, 3995 m, Lb
36d	55	15	19	**v v**	x v	x x	v v		4567 f	3996 m
34a	51	15	17	**v v**	x x	x	v	**X**	4553 f	
34b	52	16	16	**x v**	x x	x	v	**X**		3993 m
Variations	51–56	16–18	15–19						8	9

M – biarmed (x); m – male, f – female; A +St – acrocentric + subtelocentric (v); Lb – laboratory-bred voles; Pair 6/7/14 is given in capital letters to emphasize its larger sizes.

We used the karyotype of the race “Evoron” (GTG method) with the highest 2n (42) number, including 26 acrocentric (A) and 14 biarmed (M) autosomes (Kartavtseva et al. 2021) as a reference for rating and numbering chromosomes in other forms. Pairs of chromosomes were ranked not by size, but by the similarity of differential staining with those of the “Evoron” chromosomal race. The size of the Mev3 of some voles of the “Argi” race was smaller than that of the “Evoron” race; the upper arm is likely to be divided and part of the chromosome to be translocated to another chromosome, which we cannot determine at the present time.

For the tandem (TTel and TCen) and Robertsonian (Rb) fusions, two different markings of the chromosomes were used. The first marking corresponds to the tandem fusion, forming acrocentrics pairs (Mev11/19; Mev17/18 and Mev 13/15), while the second one corresponds to the Robertsonian fusion (Rb), forming metacentrics (Mev17.18 and 13.15). The different morphology possibly was the result of a centromere shift (or centromere reposition).

At least 20 chromosome plates per individual were performed to determine the number of chromosomes. The Axio Imager 1 microscope with the digital camera (AxioCamHR) and the software (Axiovision 4.7, Germany) as part of the equipment of the Joint Use Center “Biotechnology and Genetic Engineering” of the Federal Scientific Center for Terrestrial Biodiversity of East Asia, the Far East, Department of the Russian Academy of Sciences (Vladivostok, Russia) were used.

## Results

We studied two wild populations of voles (n = 17, see the Table 1) and laboratory-bred (n = 22) belonging to the Evoron vole of the chromosomal race “Argi”. We described 8 karyotypic variants which were revealed: two variants with 2n = 34, four with 2n = 36 and two with 2n = 37. Two chromosomal variants of the karyotype with 2n = 36 were found in laboratory-bred (Lb) animals (see the Table 1).

### The Karyotype variants with 2n = 36

#### Variant 2n = 36a, NF = 54

Variant 2n = 36a, NF = 54 (Fig. 3a) includes 16 biarmed and 18 acrocentric autosomes. The Mev13/15 chromosome was defined as acrocentric, while the Mev17.18 was described as a metacentric one. This chromosome is similar in size and morphology to chromosomes Mev1 and Mev2, and therefore it cannot be detected without differential staining. Mev6 and Mev 7 are always the smallest metacentric ones in this set. This variant was found in the voles from two localities; individuals from wild population No. 7 and a laboratory lineage (population No. 6).

#### Variant 2n = 36b, NF = 56

**Figure 2.**
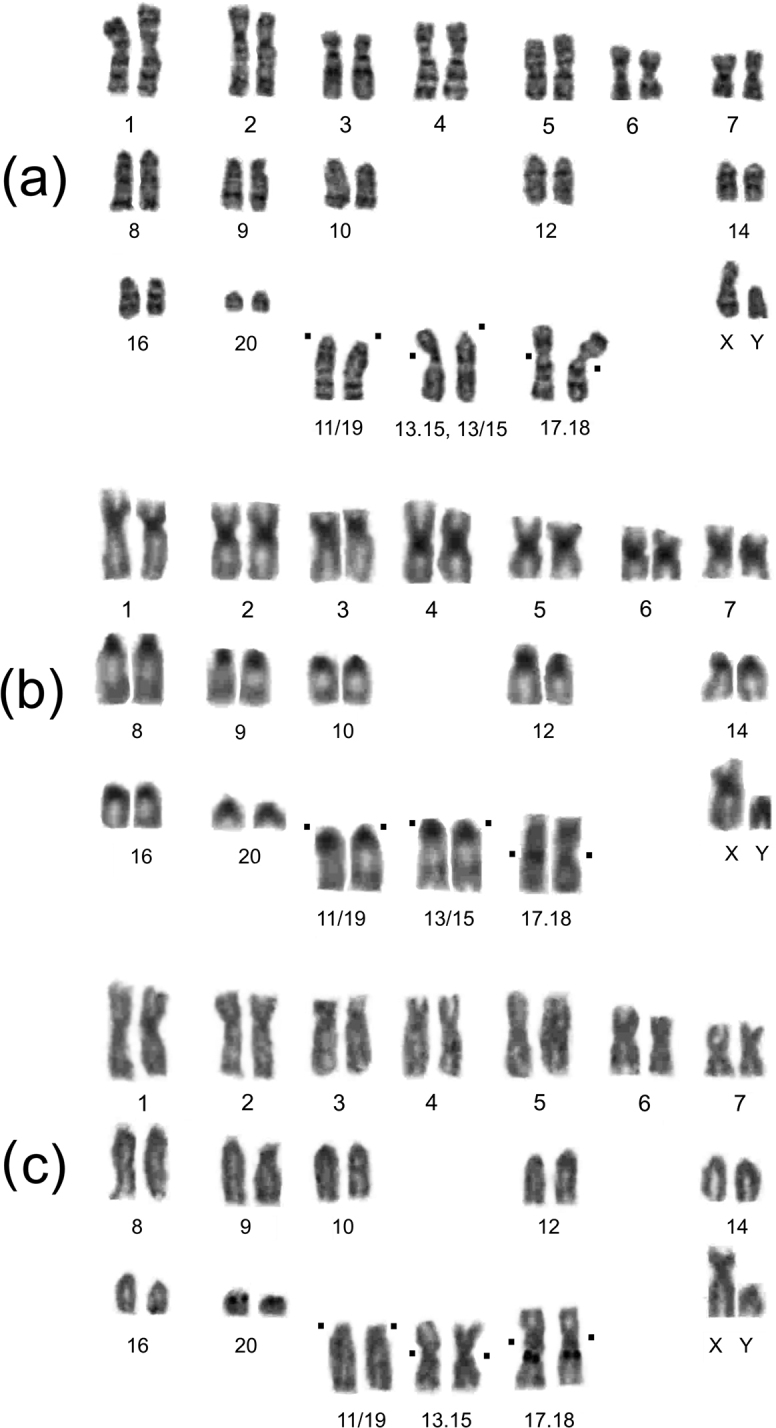
Karyotype of the *Alexandromysevoronensis* animals of the chromosomal race “Argi” with 2n = 36 **a** GTG-banded, 2n = 36c, # 4548, male from population No. 6 **b** C-banded, 2n = 36a, # 3950, male from population No. 7 **c** NORs, 2n = 36b, male of laboratory-bred from population No. 7. Black dots mark centromere positions in three pairs of chromosomes formed by the fusion of acrocentrics of the “Evoron” chromosomal race.

Variant 2n = 36b, NF = 56 contains 18 biarmed and 16 acrocentric autosomes. The Mev13.15 and Mev17.18 were defined as metacentric. This variant was found in voles of population No. 6 and laboratory lineage of population No. 7 (Fig. 2b).

#### Variant 2n = 36c, NF = 55

**Figure 3.**
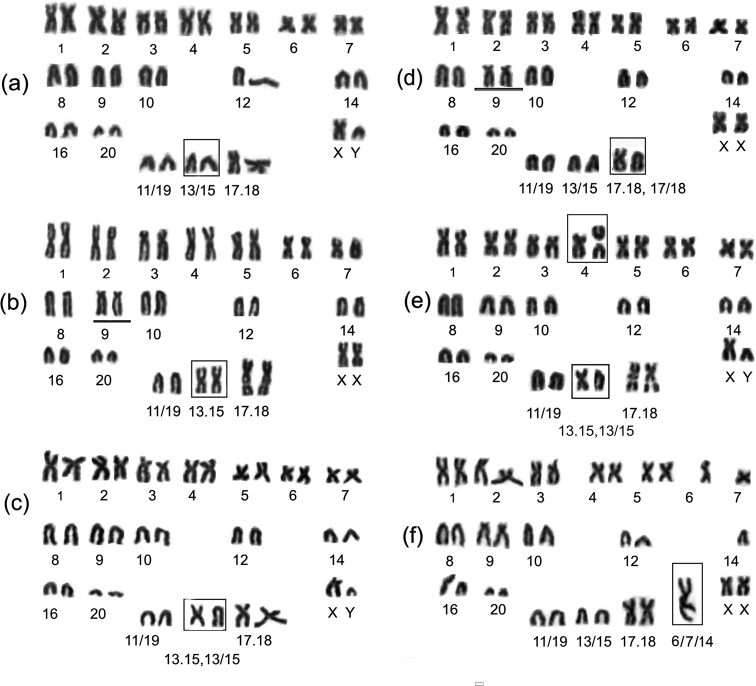
Variants of the *Alexandromysevoronensis* karyotype of the chromosomal race “Argi”. The square shows variable chromosomes **a** 2n = 36a, male, laboratory-bred from population No. 7 **b** 2n = 36b, # 4557 female from population No. 6 **c** 2n = 36c, male laboratory-bred from population No. 7 **d** 2n = 36d, # 4567 female, the square shows variable pair Mev17/18, and 17.18 **e** 2n = 37a, # 4554 male, population No. 6 the square shows rearrangement in Mev4, and heterozygous Mev13.15, 13/15 **f** 2n = 34a, # 4553 female, population No. 6, the square shows tandem translocation pairs Mev 6, 7 and 14 in heterozygous state.

Variant 2n = 36c, NF = 55 (Fig. 3c) consists of 17 biarmed and 17 acrocentric autosomes. The Mev13/15 and Mev13.15 was defined as a heteromorphic pair (A, M), and Mev17.18 was defined as a metacentric one. This variant was found in voles of both populations and in the laboratory lines.

#### Variant 2n = 36d, NF = 55

Variant 2n = 36d, NF = 55 (Fig. 3d) includes 15 biarmed and 19 acrocentric autosomes. The Mev13.15 was defined as acrocentric, and the Mev17/18 and Mev17.18 was heteromorphic (A, M). Among the majority of acrocentric, the Mev9 had clearly visible short arms (they were not accounted for when calculating the number of chromosome arms).

Most of the voles (76.5%) of the two wild populations studied had the karyotype 2n = 36, which we defined as the main karyotype for the “Argi” chromosomal race (Table 1). The differential chromosome staining performed for selected individuals made it possible to identify the chromosomes: Mev11/19; Mev13/15 and 13.15; Mev17/18 (Fig. 2) which most likely were formed due to the fusion (Rb or tandem) of six acrocentric of the ancestral karyotype for *A.evoronensis*. Heterochromatin blocks were revealed in the pericentromeric regions of all autosomes. In addition to these, the X chromosome had a centromeric block. The Y chromosome was entirely heterochromatic (Fig. 2b). The X chromosome is a medium-sized submetacentric, Y is a small acrocentric that is slightly larger than the last pair of acrocentric autosomes.

The NORs localized in the pericentromeric regions of the Mev17/18 and Mev20 of the “Argi” chromosomal race (Fig. 2c) correspond to the Mev17 and Mev20 acrocentrics of voles of the “Evoron” chromosomal race (Kartavtseva et al. 2021).

We have also found morphological variability of the chromosomes in Mev9, Mev12, and Mev16 (Fig. 3b, d). When stained for structural heterochromatin, the short arms failed to have brightly colored blocks. The variability nature of these chromosomes was not investigated due to a different spiralization.

### The Karyotype variants with 2n = 34

#### Variant 2n = 34a, NF = 51

Variant 2n = 34a, NF = 51 consists of 15 metacentric and 17 acrocentric autosomes. Both homologs of the Mev13/15 were acrocentrics (Fig. 3f). The large biarmed element Mev6/7/14 was formed by three autosomes Mev6, Mev7 and Mev14 as a result of tandem fusion. Such a karyotype was found in one female (# 4553) from a natural population No. 6.

#### Variant 2n = 34b, NF = 52

**Figure 4.**
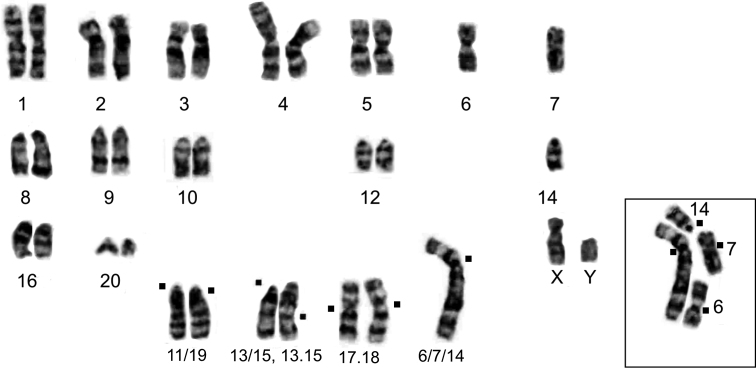
Karyotype of the *Alexandromysevoronensis* with tandem fusion of three autosomes: Mev6, Mev7, Mev14, and formation of a large biarmed element Mev6/7/14, 2n = 34b, # 3993 male. Black dots mark centromere in chromosomes involved in rearrangements.

Variant 2n = 34b, NF = 52 includes 16 metacentric and 16 acrocentric autosomes, the chromosomes Mev13/15 and Mev13.15 were heteromorphic (Fig. 4). The large biarmed element Mev6/7/14 was formed by three autosomes Mev6, Mev7 and Mev14 as a result of tandem fusion. This karyotype was found in one male (# 3993) from population No. 7.

A decrease in the chromosome number is associated with the tandem fusion of three autosomes: Mev6, Mev7, Mev14, and the formation of a large biarmed element Mev6/7/14. All four chromosomes are in a heterozygous state (Table 1). The two variants 2n = 34a (Fig. 3f) and 2n = 34b (Fig. 4) differ in the morphology of the Mev13/15 and 13.15. The variability in the size of Mev4 is possibly related to an unknown rearrangement.

Each population (Nos. 6 and Nos. 7) of the “Argi” chromosomal race revealed one individual with 2n = 34 (Table 1), the tandem fusion of autosomes Mev6, Mev7, and Mev14 as well as the formation of a large biarmed element Mev6/7/14. In the karyotype, all four chromosomes look like heterozygotes. With the Mev6/7/14 chromosome in the homozygous state, a karyotype with 2n = 32 is theoretically possible. The detection of tandem chromosome fusion in a heterozygous state in a natural population is interesting enough to be studied further focusing on understanding the DNA transformation system in telomere regions during chromosomal rearrangements. The significance of the variability of telomeric regions and their hot spots in the evolution of chromosomes was summarized in the reviews (Zhdanova et al. 2007; Baird 2018).

### The Karyotype variants with 2n = 37

#### Variant 2n = 37a, NF = 55

Variant 2n = 37a, NF = 55 (Fig. 3e) includes 16 biarmed and 19 acrocentric autosomes. The chromosomes Mev13/15 and Mev13.15 chromosomes are heteromorphic. One of the largest biarmed chromosomes has no pair. The two acrocentric chromosomes may have appeared as a result of a large biarmed chromosome fission. We could not determine the number of the arms since the strong spiralization of chromosomes did not allow us to do it. Chromosome suspensions were prepared in the field. This variant was found in population No. 6.

#### Variant 2n = 37b, NF = 55

Variant 2n = 37b, NF = 55 (see the Table 1): it consists of 16 biarmed and 19 acrocentric autosomes. The chromosomes Mev13/15 and 13.15 and Mev17/18 and 17.18 are heteromorphic. One of the large biarmed chromosomes has no pair. The two acrocentric chromosomes may have appeared as a result of a fission of a large biarmed chromosome. This variant was found in population No. 6.

This study describes Evoron voles of the “Argi” chromosomal race, with their 8 variants of the karyotype and a minimal number of chromosomes (2n = 34) as well as acrocentrics (16), as being previously not found for *A.evoronensis*. All karyotypes of voles from the two populations studied (Nos. 6 and Nos. 7) showed an acrocentric Mev11/19 to be in a homozygous state, which indicates that chromosomes had already stabilized. Chromosomes Mev13/15 and Mev13.15 in the karyotypes of both populations had a different combination (A, A; A, M; M, M), which indicates the inability of this translocation to stabilize. We can assume the possibility of the centromere repositioning as well.

After sixteen months of breeding of animals from population No. 7 in our animal facility, we got 18 litters in the first and second generations with 92 young ones from natural parents with 2n = 36 and their descendants. Twenty-two karyotyped laboratory voles (F1) with 2n = 36 showed the frequency of individuals with variable chromosome morphology of Mev13/15 and Mev13.15 (A, A; A, M; M, M) to meet a 1: 2: 1 distribution. Animals with a different chromosome number were not bred. The distribution of variants of the Mev13/15 and Mev13.15 indicates that this rearrangement has no impact on the fertility and viability of the offspring. The morphological differences in the Mev13/15 and Mev13.15 chromosomes could be explained by assuming two scenarios of chromosome fusion. The first one should be a centromere-centromere, while the second scenario should support a fusion of the centromere and telomere with different centromere inactivation, and thus forming pairs of different morphologies, as was observed for the Mev17/18 and Mev17.18 chromosomes of the “Evoron” chromosomal race (Kartavtseva et al. 2021).

## Discussion

### Comparative analysis of chromosomal rearrangements in two chromosomal races

We demonstrated that two local populations (Nos. 6 and Nos. 7) of the “Argi” chromosomal race, separated by mountain ranges (Fig. 1), have the same karyotype variants and chromosomal rearrangements. In the karyotype with 2n = 36 we always detected stabilization of Mev11/19, while chromosomal rearrangements Mev13/15 and Mev13.15; Mev17/18 and Mev17.18 had different morphology.

**Figure 5.**
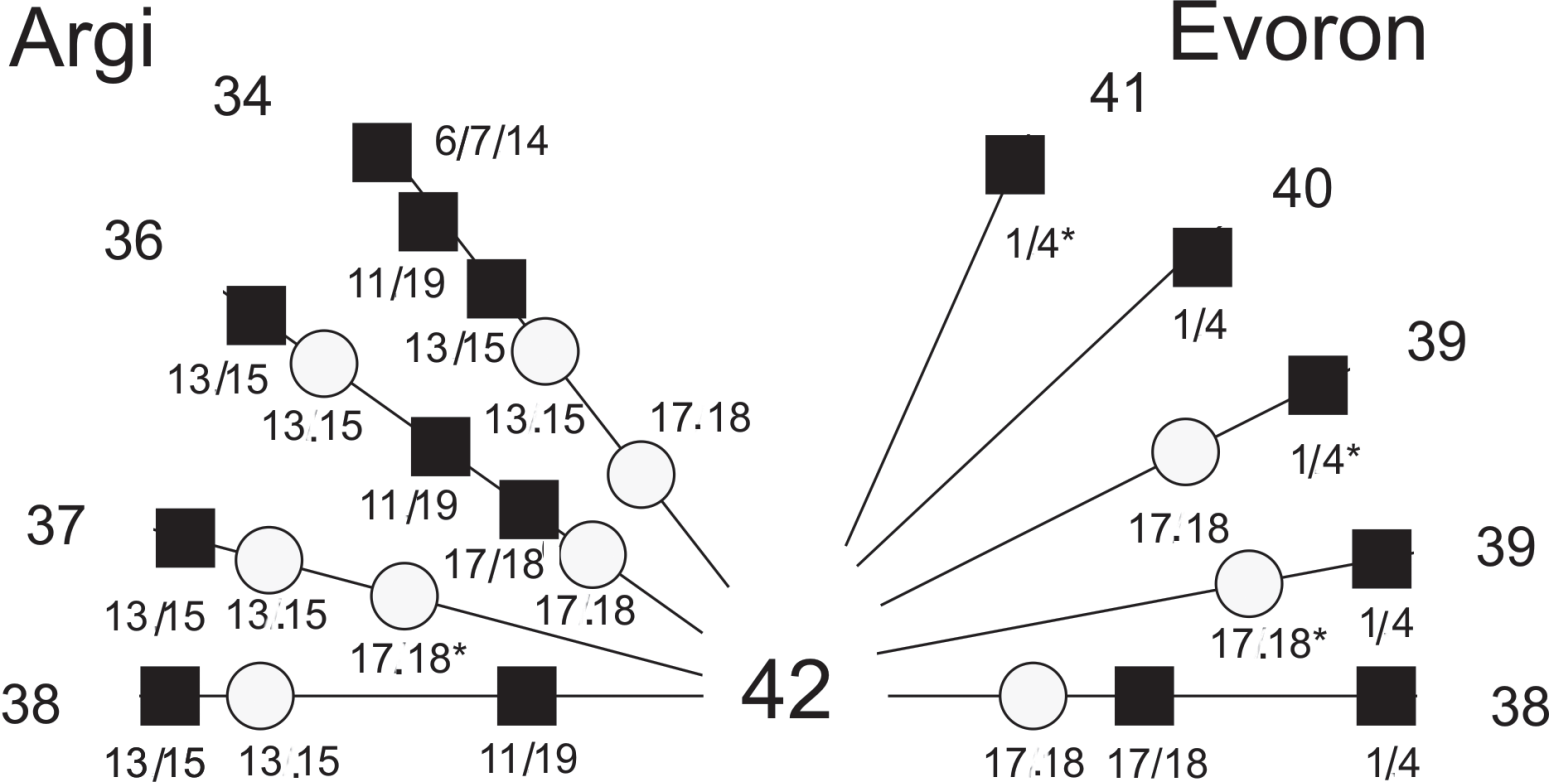
Scheme of the structural chromosomal rearrangements of the chromosome races “Argi” and “Evoron” identified in karyotypes with different numbers of chromosomes. Circles – centromeric, squares – telomeric fusion of chromosomes; asterisk – heterozygous state. The numbers on the edge of the diagram correspond to the diploid numbers found.

It is noteworthy that the 2n = 36 karyotype with these changed chromosomes exists in two geographically isolated populations, with the distance between these populations being about 500 kilometers. A recent time of divergence for *Alexandromys* was demonstrated using mt DNA data (Haring et al. 2011). The *p*-distance was small (0.0215) and matched the population level only. Two chromosomal races of *A.evoronensis*, “Argi” (2n = 34, 36, 37, NF = 51–56) and “Evoron” (2n = 38–41, NF = 54–59), differed in structural chromosomal rearrangements, which affected the number and morphology of chromosomes, as well as the number of karyotype variants (8 and 12, respectively). For example, Mev1.4 and Mev17/18 and Mev17.18 were detected for the “Evoron” chromosomal race, while Mev11.19; Mev13/15, Mev13.15; Mev6/7/14; Mev17/18 and Mev17.18 were revealed for the “Argi” chromosomal race (Fig. 5). The chromosomes Mev17.18 and Mev17/18 was present in both races.

Analysis of numerous rearrangements in mitosis and meiosis of the *Microtus* species of the Russian fauna showed that in most cases, structural rearrangements that do not affect linkage groups of important genes do not result in disruption of meiosis, nor do they serve as an obstacle to their fixation in populations. In most cases, changes revealed in centromere position are brought about by repeated chromosome fusion, with random inactivation of centromeres belonging to different chromosomes (Meyer et al. 1996). Our opinion is confirmed by the data obtained which makes us believe that the shift of the centromere (or reposition) in chromosomes formed by the fusion of chromosomes is the result of centromeres’ inactivation that have different chromosomes. Many examples of centromere repositioning occurring for other reasons in several mammalian lineages (Rocchi et al. 2012; Dobigny et al. 2017) which might be possibly described in voles.

### Group “maximowiczii”

The range of voles with an ancestral karyotype (2n = 42) could cover the area from Lake Baikal to the coast of the Sea of Okhotsk in the eastern part of Siberia. According to Pozdnyakov (1996), fluctuations in the climatic conditions of this territory can lead to a significant change in the ranges of many species, including voles. Paleogeographic reconstructions also confirm the representatives of the “maximowiczii” voles (Golenishchev 1982): *A.maximowiczii* (Schrank, 1859), *A.mujanensis* (Orlov et Kovalskaya, 1978), *A.evoronensis* to belong to the boreal subcomplex of the mammoth theriocomplex (Baryshnikov and Markova 2009; Erbajeva et al. 2011) which included vast areas of tundra and meadow steppes during the cold period of the Late Pleistocene in Eurasia. With the beginning of landscape changes in the Holocene, this environment of tundra and meadow steppes decreased, but could be preserved in refugia.

For example, in the Middle Holocene, 7–5 thousand years ago, peculiar meadow steppes were widespread in the intermountain basins of Northern Transbaikalia (Mikheev 1974), very close to the habitats of *A.mujanensis* and *A.evoronensis*. Late Holocene cooling caused severe changes in vegetation (Neustadt 1957; Bazarov 1968; Mikheev 1974), the disappearance of areas of meadow steppes and, as a result, the reduction of the vole range, its division into parts and the disappearance of species over a wide range. There are no paleontological data on these species, but there are data on *A.maximowiczii*, morphologically close and little distinguishable from *A.mujanensis* and *A.evoronensis* which appeared in the territory of Transbaikalia (Erbajeva 1970; Erbajeva et al. 2011) and the South of the Russian Far East in the Late Pleistocene and Early Holocene (Alekseeva and Golenishchev 1986; Voyta et al. 2019). The vole *A.mujanensis* has intra- and interpopulation variability in the morphology of four pairs of chromosomes (2n = 38; NF = 50–53) (Lemskaya et al. 2015; Kartavtseva et al. 2019).

According to Pozdnyakov (1996), *A.mujanensis* and *A.evoronensis* could have appeared simultaneously, as *A.mujanensis* in Buryatia and Transbaikalia, and *A.evoronensis* in the south of the Russian Far East. If we talk about the rate of chromosomal transformations, our data makes it possible to conclude that five structural rearrangements occurred in the karyotype of the vole *A.evoronensis* over a short geological period. The number of rearrangements from the ancestral karyotype to the *A.mujanensis* karyotype has not been determined, but there should be no less than two of them.

*A.maximowiczii* karyotype also underwent structural and intrachromosomal rearrangements (Meyer et al. 1996; Kartavtseva et al. 2008). FISH methods revealed intrachromosomal rearrangements in the syntenic regions of several chromosomes (the number of chromosome pairs is unknown) in *A.maximowiczii* (2n = 44), *A.mujanensis* (2n = 38), and *A.evoronensis* (2n = 36) (Romanenko et al. 2018). The authors suggest that intrachromosomal rearrangements in syntenic regions of chromosomes possibly serve as the main evolutionary force modulating the genome architecture. It should also be noted that the study used an *A.evoronensis* specimen (2n = 36) belonging to population No. 6 of the “Argi” chromosomal race, whose karyotype was previously published without differential staining (Sheremetyeva et al. 2017a). We also believe that the FISH analysis of three chromosomal polymorphic species used a pair of chromosomes with a stable morphology; otherwise, the existing differences in chromosomal rearrangements could be attributed not to the listed species differences, but to intraspecific variability.

Previously, for ten Palearctic vole species (whose chromosome number varied from 30 to 50), comparative G-banding and chromosome staining with specific *Microtusagrestis* (Linnaeus, 1961) revealed chromosomal rearrangements that distinguish this species from its ancestral karyotype (2n = 54) (Lemskaya et al. 2010). The greatest number of fusion / fission rearrangements was observed in two species of the genus *Alexandromys* Ognev, 1914 (= *Microtus*) – *A.oeconomus* (2n = 30) and *A.maximowiczii* (2n = 41) (Lemskaya et al. 2010). Based on karyological data and data on the average time of appearance of the *Microtus* sensu lato species, the stabilization of 6 chromosomal rearrangements was established, leading to a change in the number of *A.oeconomus* chromosomes of greater than once every million years of evolution, while, for other rodents this process takes more than 1 million years.

On average, one rearrangement of this type (fusion / fission) was believed to occur once every million years (Schibler et al. 1998). The second studied vole species, *A.maximowiczii* has not had its number of such rearrangements determined yet, though at least nine chromosome fusions of the ancestral karyotype have been shown to bring about the formation of a karyotype with 2n = 41 (Lemskaya et al. 2010).

The chromosome painting data that are now available for many species from different orders (Murphy et al. 2001; Ferguson-Smith and Trifonov 2007) help to estimate the average rate of evolutionary rearrangements during different periods in distinct lineages and propose two main modes of karyotype evolution rate: an ancestral slow rate (one or less exchange per 10 mya) and higher rates. Record high rates of karyotype evolution were found in muroid rodents, canids, gibbons and equids (reviewed in Ferguson-Smith and Trifonov 2007). Chromosome rearrangements are central to studies of genome evolution, as our understanding of the evolutionary consequences of the early stages of karyotypic differentiation (i.e. polymorphism), especially the non-meiotic impacts, is surprisingly limited (Dobigny et al. 2017). All known chromosomal rearrangements can be involved in intra- and interpopulation polymorphism in mammals, especially in evolutionarily young species, but tandem fusions are the most deleterious mutations (King 1993; Dobigny et al. 2017). Tandem fusions (i.e. centromere–telomere, or telomere–telomere fusions (Elder and Hsu 1988) are considered as highly deleterious rearrangements because heterozygous carriers were displaying at least a 50% decrease in the production of balanced gametes (White 1973; King 1993). The tandem fusions were detected in a heterozygous state in three mammal species only (Dobigny et al. 2017) in *Urodermabilobatum* bats (Owen and Baker 2001), South American rodent tuco-tuco *Ctenomystalarum* (Massarini et al. 2002) and in one of southern birch mouse *Sicistasubtilis* (Kovalskaya et al. 2011). Later we added three more species to this list (Kartavtseva et al. 2021): Arctic foxes *Alopexlagopus* (Radzhabli and Grafodatskii 1977), voles *Alexandromysmaximowiczii* (Meyer et al. 1996; Kartavtseva et al. 2008), and *A.evoronensis* (chromosomal race “Evoron”) (Kartavtseva et al. 2021). The study of the “Evoron” chromosomal race revealed a heterozygous state of tandem fusion (Mev1/4) with a frequency of 0.47. For the “Argi” chromosomal race, the frequency of a heterozygous state of tandem fusion (Mev6/7/14) is 0.12 (see the Table 1).

Structural chromosomal rearrangements of “maximowiczii” voles in Asia (*A.maximowiczii* and *A.evoronensis*), as well as species of the genus *Microtus* in Europe, occurred in isolated mountain populations during the Late Pleistocene and Holocene climate change. Studies of karyotype transformation in various species allow us better to understand the role of chromosomal rearrangements in speciation.

## Conclusions

Thus, we demonstrated two isolated populations of the “Argi” chromosomal race to have identical polymorphism (2n = 34, 36, 37, NF = 51–56). We revealed the multiple chromosomal rearrangements with the tandem fusions (Mev11/19, Mev13/15, Mev17/18, Mev6/7/14) and the Robertsonian translocations (Mev13.15 and Mev17.18) that led to eight new variants of the karyotype described. We observed the tandem fusion (Mev6/7/14) of chromosomes in heterozygous states in both populations.

For *A.evoronensis*, the variation in the number of chromosomes exceeded the known 2n = 34, 36 up to 2n = 34, 36, 38–41. The combination of all the variations of chromosomes for the species made it possible to describe 20 variants of the *A.evoronensis* karyotype, with 11 chromosomes which being involved in multiple structural rearrangements. In the “Evoron” chromosomal race 4 chromosomes (Mev1, Mev4, Mev17, and Mev18) and in the “Argi” chromosomal race 9 chromosomes (Mev6, Mev7, Mev14, Mev13, Mev11, Mev15, Mev17, Mev18, and Mev19) were observed. Tandem and Robertsonian rearrangements (Mev17/18 and Mev17.18) were revealed in both “Evoron” and “Argi” chromosomal races.
